# Geochemical Study of Bitumen Residues on Potsherds from the al-Qusur Monastery (7th–9th c. CE): Composition and Origin

**DOI:** 10.3390/molecules30092006

**Published:** 2025-04-30

**Authors:** Jacques Connan, Julie Bonnéric, Rémi Perrogon, Michael H. Engel, Renaud Gley, Alex Zumberge, Philippe Schaeffer

**Affiliations:** 1 Université de Strasbourg, 23 rue Antoine de Saint-Exupéry, 64000 Pau, France; 2 French Research Centre in the Arabian Peninsula, USR3141, Kuwait City 92438, Kuwait; bonneric.julie@gmail.com; 3 LA3M-UMR 7298, Aix-Marseille University, 13100 Aix-en-Provence, France; remi.perrogon@gmail.com; 4 School of Geosciences, The University of Oklahoma, 100 East Boyd Street, Norman, OK 73019-1009, USA; ab1635@ou.edu; 5 Laboratoire Interdisciplinaire des Environnements Continentaux, UMR7360, Site Chamois, 15 Avenue de Chamois, 54500 Vandeouvre-les-Nancy, France; renaud.gley@univ-lorraine.fr; 6 GeoMark Research Inc., 9748 Whithorn Drive, Houston, TX 77095, USA; azumberge@geomarkresearch.com; 7 Institut Le Bel, UMR 7177, Université de Strasbourg, 4 rue Blaise Pascal, 67000 Strasbourg, France; p.schaef@unistra.fr

**Keywords:** Kuwait, Failaka Island, al-Qusur, monastery, potsherds, bitumen, biomarkers, steranes, terpanes, carbon and hydrogen isotopes, geochemical analysis, oil seeps, Iran

## Abstract

Geochemical and isotopic analysis of bitumen lining potsherds from the al-Qusur monastery (second half of the 7th c. CE and the middle of the 9th c. CE), at the central part of Failaka Island (Kuwait Bay), confirms the presence of two distinct compositional categories that can be matched to contemporary sources from two different areas of Iran: the Kermanshah province on one side, and the Khuzestan–Fars–Busher provinces on the other side. Potsherds comprise different types: TORP-S amphorae, TORP-C amphorae, SPORC storage jar, turquoise alkaline-glazed jar (TURQ.T), and CREAC jar. There is no relationship between the type of potsherd and the origin of bitumen. The bitumen coating SPORC jar, first identified as a kind of juice strainer to filter the «garum-like juice», was examined in greater details to try to identify traces of fish sauce mentioned in the Arabic kitchen books as ‘*murri’*, and quite similar to the Roman *garum.* The mineralogical analysis exhibits the classical minerals of archaeological mixtures (quartz, calcite, dolomite) and no halite. Hydrocarbons, alcohols, and methyl esters show a typical biodegraded bitumen signature but no fatty acids and terpenoids. It seems that the bitumen matrix has not adsorbed any molecules from the presumed «*garum*» filtered in the basin.

## 1. Introduction

**Figure 1:**
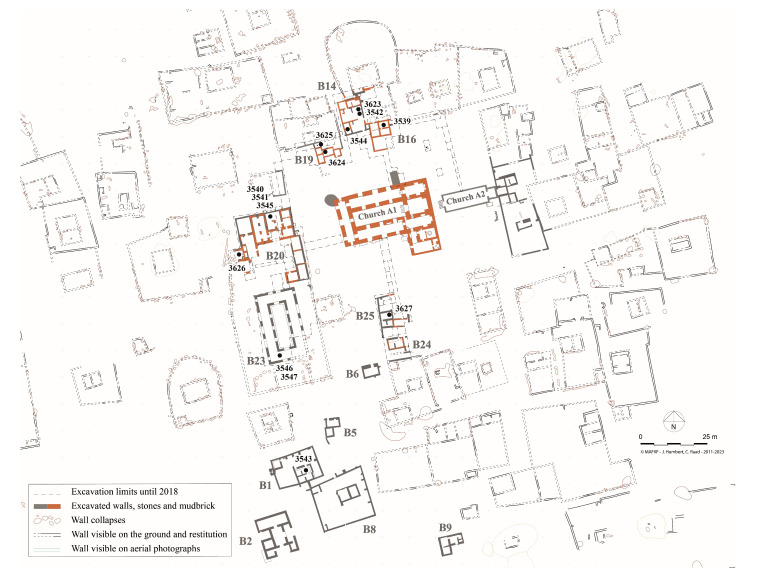
Location of the bitumen samples in al-Qusur monastery (J. Humbert, C. Raad, © MAFKF).

The bitumen samples originated from the site of al-Qusur, located in the middle of Failaka Island, c.19 km from the southern tip of Kuwait Bay. The central part of al-Qusur was identified by the French-Kuwaiti Archaelogical Mission in Failaka (MAFKF) as a monastery mainly occupied from the second half of the 7th c. CE to the middle of the 9th c. CE. The al-Qusur monastery, at the central part of Failaka Island, has been excavated by the French-Kuwaiti Archaeological Mission in Failaka (MAFKF) since 2012. The results of the past campaigns have been published [1,2,3,4], authenticating the monastic nature of the site and dating its main occupation from the second half of the 7th c. CE and the middle of the 9th c. CE. The site was first excavated by the Archaeological Mission in the Arabian Gulf in 1975–1976, then the French Mission in Kuwait in 1988–1989 and 2007–2009, the Kuwaiti-Polish Archaeological Mission in 2011 and 2013, and is still being studied today by the French-Kuwaiti Archaeological Mission in Failaka, since 2011, and the Kuwaiti-Slovak Archaeological Mission in 2007. In spite of the number of excavation seasons carried out on the site by many teams, there is always a debate about the interpretation of the whole site. If the center of al-Qusur is clearly a monastery, as evinced by the discovery of a monumental church (building A1), a refectory (building B23), and at least one monk’s cell (building B16), it is still to be determined if the periphery of the site is part of the monastery or not (Figure 1). One of the current hypotheses is that the central part of the monastery (c. 0.78 ha) could be a *coenobium* surrounded by scattered cells accommodating monks wishing to live more isolated from the community. Following this hypothesis, the monastery would be covering c. 335 ha and be accurate with the rules of Abraham of Kashkar, promoting semi-cenobitic types of monasteries.

Al-Qusur was part of a network of monasteries associated with the Church of the East that spread across what is now Iraq, Iran, and the Gulf. Not only monks traveled between these areas but also goods. For instance, most of the pottery used in al-Qusur was made in Iraq and Iran. One of the aims of the French-Kuwaiti Archaeological Mission in Failaka is to study these exchanges and to understand how the monastery was supplied. For this reason, geochemical and isotopic analyses of nine samples of bituminous mixtures coating potteries were carried out. The purpose of this study was to document the composition of the bituminous mixture and to find the source of the bitumen.

## 2. Materials and Methods

### 2.1. Bitumen at al-Qusur

In al-Qusur, no bitumen had yet to be shown to be used for building construction. However, in building B20, a food processing building, bitumen was found to be associated with pottery in cooking areas. It mainly appears on pottery jars, as inside coating to waterproof them. One Syriac inscription on a sherd may also have been written with this material. The main documented use of bitumen is as coating on the inside of the pottery vessels. The jars do not appear to have been coated with bitumen on the site, but were probably imported already coated. However, it is worth mentioning that bitumen might be supplied by the nearby and contemporary settlement of al-Qurainiyah, on Failaka’s northern coast, where the Kuwaiti-Italian Archaeological Mission reported remains that could be a bitumen production workshop [5]. The bitumen might have been delivered there in solid form before being heated and used in the architecture or maintenance of boats [5].

### 2.2. Archaeological Samples Analyzed

**Figure 2:**
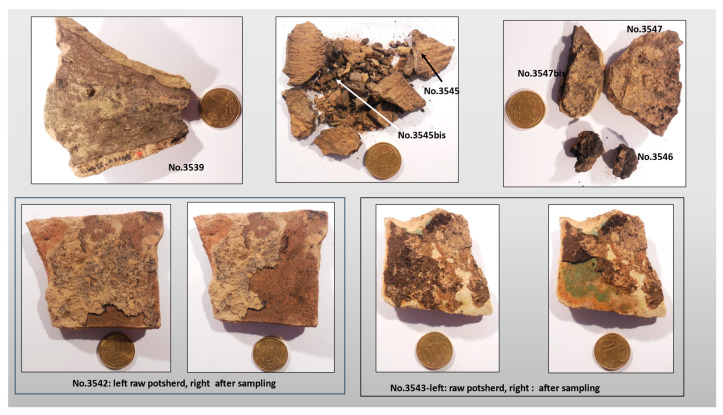
Picture of eight samples of al-Qusur showing various aspects of the bitumen coating the interior face of potsherds. Some pictures show the situation before and after sampling.

Thirteen bituminous samples were selected and a total of fifteen geochemical analyses (two samples were analyzed with two aliquots) were undertaken. Initial information (date, archaeological reference, context) including the type of potsherd is listed in Table 1. On potsherds, the coating of the interior face may be very thin as on the bottom of the torpedo jar No.3539, or more extensive as in samples No.3542 and No.3543 (Figure 2). The bituminous mass may also show some imprints (ropes?) as in sample No.3545 (Figure 2). The quantity of bituminous mixture, collected from a torpedo jar (TORP-C), suggests that it was a bituminous mixture stored in the jar. This sample was studied with several aliquots: No.3546, 3547 and 3547bis (Figure 2).

The ceramic categories that were sampled are part of the same group of sandy buff wares, sharing a similar clay composition with a rather fine calcareous ground mass, fine quartz elements, and small magmatic rocks. There are two categories of amphorae (Torpedo -S and -C) with an internal bitumen coating, known as *torpedo jars* because of their profiled shape. The samples No.3542, 3544, 3623, and 3624 were collected on sherds of TORP-S amphorae belonging to the Late Sassanian, Early Islamic assemblages (5th–8th c. CE). This type is found in abundance in the region, but is gradually replaced by lighter-paste amphorae (TORP-C) during the 8th century [6]. The samples No.3539, 3546, 3547, 3625, and 3626 are related to the latter type. It is contemporaneous to large storage jars (SPORC), such as sample No.3545. This type is quite rare in the area and has some parallels with material from the coastal site of Siraf [7]. The main centers of origin for these categories are supposedly located around the urban centers of central and southern Mesopotamia, from Susa (Kermanshah province, Iran) to al-Hira (Najaf district, Iraq) and Basra province (Iraq). A TORP-S production kiln has been identified in Telūl Hamediyāt, northeast of Baghdad (Hamrin basin, Diyala province, Iraq) and dated from the 6th–7th century [8,9]. This workshop was located alongside a wine-production facility and the amphorae were apparently potted on the site. This could seem to support the hypothesis that ceramics were produced close to the agricultural area in the Mesopotamian basin, instead of near the source of bitumen production in southwest Iran [10].

Sample No.3543 was recovered from a sherd of a turquoise alkaline-glazed small jar (TURQ.T). The layer of bitumen covers a large part of the internal and external surfaces and seems to have accumulated particularly on the shoulder and neck of the object. The various traces of dripping and the absence of repair marks suggest that this object was used to contain and handle bitumen in liquid form. Although turquoise ceramics were used over a long period of time, the forms found at the al-Qusur site correspond more closely to the types identified at the Sir Bani Yas site and dated mainly between the mid-7th and mid-8th century [11,12]. Based on the clay and glaze used, the area of origin of these products is probably in southern Mesopotamia, in the Basra region. Further archaeometrical analyses of the composition of the glazes should make it possible to identify even more precisely the regions of production [13].

Samples No.3540 and No.3541 are extracted from a ceramic category that is difficult to precisely identify. In terms of clay and surface treatment, this jar is similar to those made at Siraf on the Iranian coast, known as CREAC, and dated between the late 8th and the 10th century [12]. It is possibly another sandy buff ware belonging to the same group as TORP, TURQ, and SPORC, but with an overfired body, with a large number of magmatic rock inclusions within the clay matrix.

### 2.3. Analytical Procedures

#### 2.3.1. Bitumen Analysis

All archaeological and geological samples were subjected to the same analytical procedure conducted at GeoMark Research Ltd. (Houston, TX, USA). The ground potsherd samples were Soxhlet extracted with a mixture of 372 mL dichloromethane and 28 mL methanol. The dried dichloromethane extracts were deasphalted using *n*-hexane. The deasphalted fraction was separated into saturated hydrocarbons, aromatic hydrocarbons, and resins using gravity flow column chromatography employing a 100–200 mesh silica gel support activated at 400 °C prior to use. Hexane was used to elute saturates, dichloromethane to elute the aromatic hydrocarbons, and dichloromethane/methanol (50:50 *v/v*) to elute the resin (NSO) fraction. Following solvent evaporation, the recovered fractions were quantified gravimetrically. The C_15+_ saturated hydrocarbon fraction was subjected to molecular sieve filtration (silicalite, Union Carbide S-115 powder) after the technique described by West et al. [14]. An aliquot of the total alkane fraction was not fractionated by silicalite in order to preserve access to the *n*-alkanes.

Gas Chromatography-Mass Spectrometry (GC-MS) analyses of C_15+_ branched/cyclic and aromatic hydrocarbon fractions (in order to determine sterane and terpane biomarker distributions and quantities) and aromatic hydrocarbon fractions were performed using an Agilent 7890A or 7890B GC interfaced to a 5975C or 5977A mass spectrometer (Wilmington, DE, USA). The J&W HP-5 column (50 m × 0.2 mm; 0.11 µm film thickness) is temperature programmed from 150 °C to 325 °C at 2 °C/min for the branched/cyclic fraction and 100 °C at 3 °C/min for the aromatic fraction. The mass spectrometer is run in the selected ion mode (SIM), monitoring ions *m*/*z* 177, 191, 205, 217, 218, 221, 231, and 259 for branched and cyclic alkanes, and *m*/*z* 133, 156, 170, 178, 184, 188, 192, 198, 231, 239, 245, and 253 for aromatics. In order to determine absolute concentrations of individual biomarkers, a deuterated internal standard (d4-C29 20R Ethylcholestane, Chiron Laboratories, Norway) is added to the C_15+_ branched/cyclic hydrocarbon fraction and a deuterated anthracene standard (d10) is added to the aromatic hydrocarbon fraction. Response factors (RF) were determined by comparing the mass spectral response at *m*/*z* 221 for the deuterated standard to hopane (*m*/*z* 191) and sterane (*m*/*z* 217) authentic standards. These response factors were found to be approximately 1.4 for terpanes and 1.0 for steranes. Concentrations of individual biomarkers on the branched/cyclic fraction were determined using the equation below:

Conc. (ppm) = [(ht.biomarker)ng standard)]/[(ht.standard) (RF) (mg b/cy fraction)]

RF = response factor, ng = nanograms, for branched and cyclic alkanes (b/cy) peak heights (ht) are used, for aromatics the same equation is used with peak areas.

The C_15+_ saturates, C_15+_ aromatics, asphaltenes, and resins were analyzed for their respective carbon isotope (δ^13^C in ‰/VPDB = Vienna Pee Dee Belemnite ) compositions. The asphaltenes and resins were also analyzed for their stable hydrogen isotope compositions. Details for all of the methods for stable isotope analyses are described in Connan et al. [15].

#### 2.3.2. Mineralogical Analysis

All samples were ground prior to the XRD analysis. The mineralogy was determined using a D8 Discover Bruker diffractometer (Bruker, Karlsruhe, Germany) equipped with an X-ray tube emitting Cu-Kα1 radiation (λ = 1.5406 Å) at 40 kV and 40 mA. X-ray diffraction patterns were recorded with a LynxEye detector (Bruker) under ambient conditions and from 2.5° to 65° 2θ, with a step size of 0.035° 2θ, and with a calculation time of 3 s per step. Analyses were performed using DIFFRAC.EVA Bruker software (Version Eva, Release 2025, 7.3.0.2 (64 Bits) access via Eva 3) and diffraction peaks were identified using the powder diffraction file database.

#### 2.3.3. Complementary Organic Analyses

An aliquot of the sample No.3545 was extracted by a mixture of CH_2_Cl_2_/MeOH 1:1 (*v*/*v*) and then acetylated (Ac_2_O/Pyr) and methylated (CH_2_N_2_). The mixture was subsequently fractionated by silica gel chromatography into two fractions: an apolar one (F1) containing the hydrocarbons and a more polar one (F2) containing the alcohols (as acetates) and the fatty acids (as methyl esters).

##### GC-FID 

GC analyses were carried out on a Hewlett Packard 6890 gas chromatograph (Agilent Technologies Inc., Wilmington, DE, USA) equipped with an on-column injector used in the “track-oven” mode, a flame ionization detector, and an HP-5 fused silica capillary column (30 m × 0.25 mm; 0.25 μm film thickness). H_2_ was used as carrier gas (constant flow mode, 2.5 mL/min), and the oven was programmed as follows: 70 °C (5 min), 70–200 °C (4 °C/min), 200–300 °C (10 °C/min), isothermal at 300 °C.

##### GC/MS

GC/MS analyses were performed on a Thermo GC Trace gas chromatograph (Thermo Fisher Scientific, Waltham, MA, USA) equipped with an HP5 MS column (30 m × 0.25 mm × 0.1 µm) and a temperature-programmable injector linked to a Thermo TSQ Quantum mass spectrometer (Thermo Fisher Scientific). Helium (constant flow, 1.2 mL/min) was used as carrier gas and the oven was programmed as follows: 70 °C (5 min), 70–200 °C (4 °C/min), 200–300 °C (10 °C/min), 40 min isothermal at 300 °C. The mass spectrometer was operating in electron impact (EI) mode at 70 eV with a scan range of *m/z* 50–700. 

## 3. Results and Discussion

### 3.1. Gross Composition of Extractable Organic Matter

**Figure 3:**
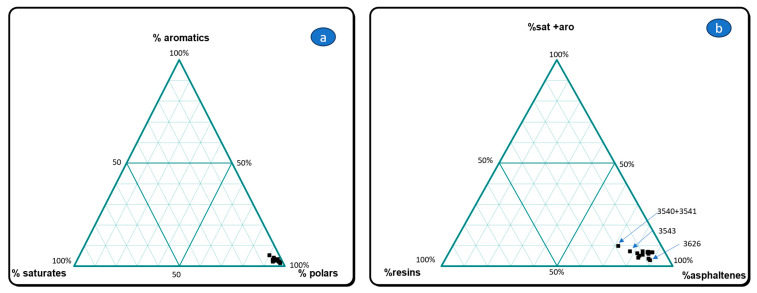
Gross composition of the dichloromethane extract in two ternary diagrams: (**a**) % saturates vs. % aromatics vs. % polars and (**b**) % sat + aromatics vs. % resins vs. % asphaltenes.

Gross composition data are listed in Table 2. The plot of % HC (sat + aro) vs. % resins (NSO’s) vs. % asphaltenes (Figure 3b) and % saturates vs. % aromatics vs. % polars (Figure 3a) shows that bituminous mixtures of al-Qusur possess the characteristic composition of archaeological bitumens: high amounts of polars (resins + asphaltenes) and asphaltenes and low quantities of hydrocarbons and resins. One may notice some slight differences among samples in Figure 3b.

### 3.2. Isotopic Compositions

**Figure 4:**
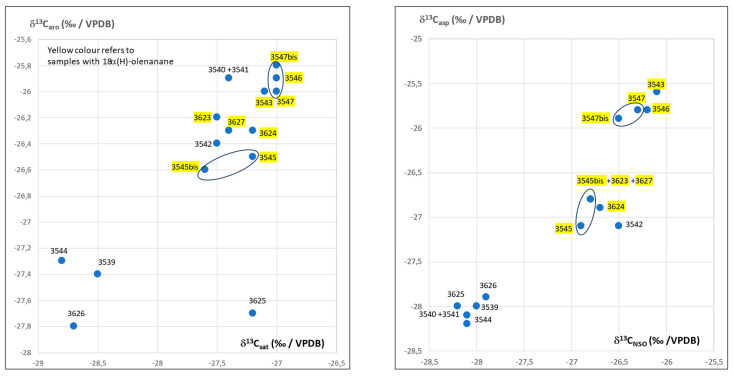
Plot of δ^13^C (‰/VPDB) of aromatics vs. δ^13^C (‰/VPDB) of saturates and δ^13^C (‰/VPDB) of asphaltenes vs. δ^13^C (‰/VPDB) of NSO for the al-Qusur samples.

Isotope data are listed in Table 2. The plot of δ^13^C_sat_ (‰/VPDB) vs. δ^13^C_aro_ (‰/VPDB) and δ^13^C_asp_ (‰/VPDB) vs. δ^13^C_NSO_ (‰/VPDB) in Figure 4 reveals at least two main types of bitumen. One may notice a good match in duplicate samples (No.3545 and 3545bis and 3547 and 3547bis, Figure 4).

**Figure 5:**
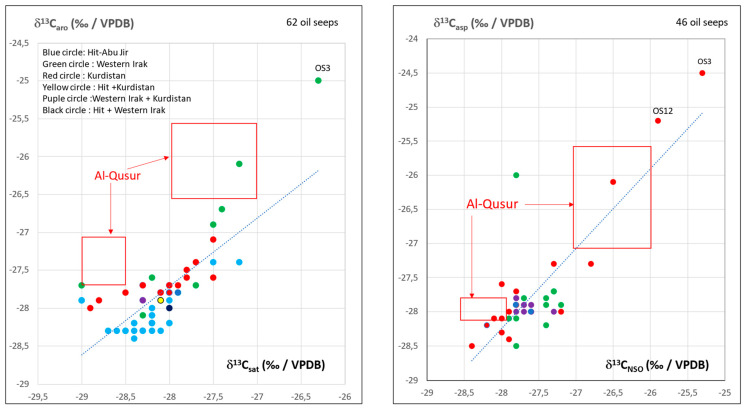
Plot of δ^13^C (‰/VPDB) of aromatics vs. δ^13^C (‰/VPDB) of saturates and δ^13^C (‰/VPDB) of asphaltenes vs. δ^13^C of NSO for the 62 and 46 oil seeps of Iraq: comparison with al-Qusur’s data.

**Figure 6:**
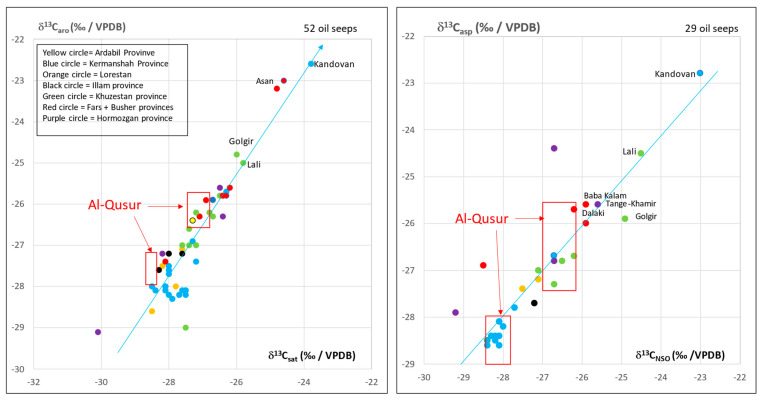
Plot of δ^13^C (‰/VPDB) of aromatics vs. δ^13^C (‰/VPDB) of saturates and δ^13^C (‰/VPDB) of asphaltenes vs. δ^13^C of NSO for 52 and 29 oil seeps of Iran: comparison with al-Qusur‘s data.

Comparison of the al-Qusur data with data collected on oil seeps from Iraq (Figure 5) and Iran (Figure 6) shows that the bitumen of al-Qusur matches the oil seeps of Iran, but not those of Iraq.

**Figure 7:**
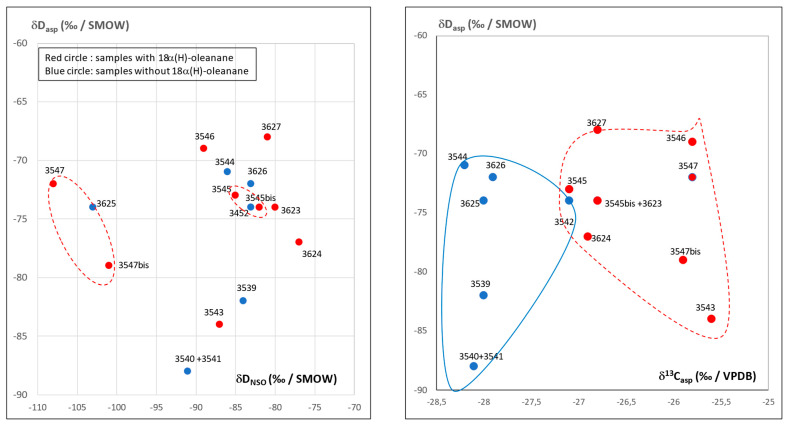
Plot of the δD (‰/VSMOW) of asphaltenes vs. δD (‰/VSMOW) of resins (NSO) and δD (‰/VSMOW) of asphaltenes vs. δ^13^C (‰/VPDB) of asphaltenes with the identification of samples containing 18α(H)-oleanane.

One group of bitumen is originating from Kermanshah province whereas the second group is likely coming from the Khuzestan–Busher–Fars provinces. The plot of δD_asp_ (‰/VSMOW) vs. δD_NSO_ (‰/VSMOW) in Figure 7 shows values (−90 to −70 ‰/VSMOW) for asphaltenes which are currently reported for archaeological bitumens. No relationship exists between the origin of bitumen and δD values (Figure 7). This situation is usual for δD parameter is not a source parameter but rather reflects the state of bitumen alteration which is not very intense here [15,16]. Values higher than −100 (‰/VSMOW) in resins of samples No.3547 and No.3547bis reflect even a well-preserved fraction [17]. One may note the good match between the values for samples No.3547 and No.3547bis as well as for samples No.3545 and No.3545bis (Figure 7) which documents the reliability of the δD values.

### 3.3. Biomarker Analysis

**Figure 8:**
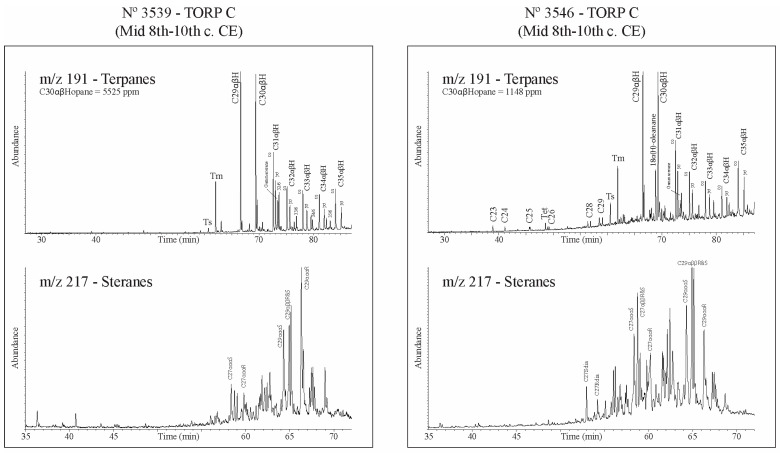
Mass fragmentograms of steranes (*m*/*z* 217) and terpanes (*m*/*z* 191) of sample No.3529 without 18α(H)-oleanane and sample No.3546 with 18α(H)-oleanane. Significance of abbreviations: C_29_αβH = norhopane, C_30_αβH = 17α,21β-hopane, C_31_αβHR = 17α,21β,22R-30-homohopane, C_27_αββR+S = 5α,14β,17β-20R+20S-cholestane, C_28_αββR+S = 5α,14β,17β-20R+20S-24methylcholestane, C_29_αββR+S = 5α,14β,17β-20R+20S-24ethylcholestane, C_29_ααα20S = 5α,14α,17α-20S-24ethylcholestane, Ts = 18α-22,29,30-trisnorneohopane, Tm = 17α-22,29,30-trisnorhopane, C_34_αβHS = C34-17α,21β-22S-extended hopane.

Mass fragmentograms of steranes (*m*/*z* 217) and terpanes (*m*/*z* 191) of two torpedo C potsherds are presented in Figure 8.

Terpanes of sample No.3539 show a high Tm/Ts ratio, a moderate gammacerane content, the complete hopane family, and the occurrence of the hexahydrobenzohopane series (C_32_/6 to C_35_/6). The tricyclopolyprenanes are missing (water washing? biodegradation? origin?). The steranes are biodegraded with a classical pattern (C_27_ < C_28_ < C_29_). No selective degradation of C_29_αααR is seen.

Terpanes of sample No.3546 also have a high Tm/Ts ratio but the outstanding feature is the occurrence of 18(α)H-oleanane, characteristic of oils from Khuzestan, Busher and Fars [18]. In this sample, tricyclopolyprenanes (C_23_/3 to C_29_/3) and hexahydrobenzohopanes (C_32_/6 to C_35_/6) are present. Steranes are not biodegraded and as expected with this type of chemistry, contain diasteranes (C_27_S and C_27_R dia). The occurrence of 18α(H)-oleanane, identified on samples in Figure 4 and Figure 7, confirm the existence of at least two main groups of bitumens as already expected on the basis of δ^13^C data.

Biomarker data are listed in Table 3.

**Figure 9:**
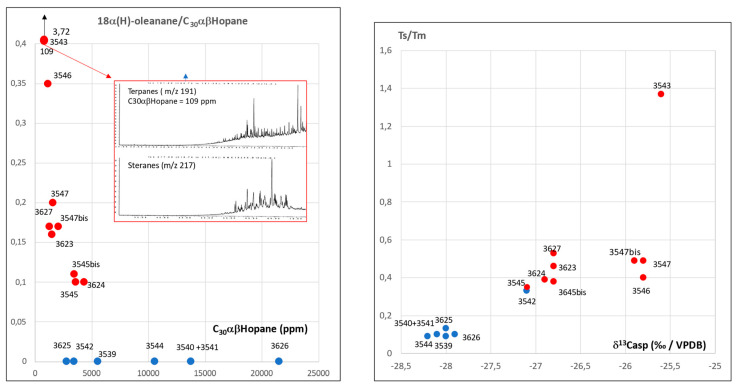
Plot of 18α(H)-oleanane/C_30_αβHopane vs. C_30_αβHopane (ppm) and Ts/Tm vs. δ^13^Casp (‰/VPDB) with the al-Qusur samples.

**Figure 10:**
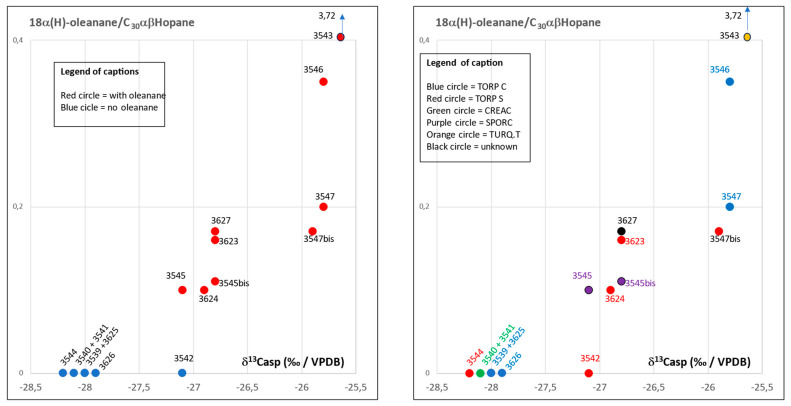
Plot of 18α(H)-oleanane/C_30_αβHopane vs. δ^13^Casp (‰/VPDB) in samples of al-Qusur with and without the identification of type of potsherds.

The plot of Ts/Tm vs. δ^13^Casp (‰/VPDB) and 18α(H)-oleanane vs. C_30_αβHopane (ppm) in Figure 9 confirms the distribution of the samples into two main groups: samples No.3543, 3546, 3547, 3547bis, 3545, 3545bis, 3627, 3623, and 3624 on one side, samples No.3539, 3540 + 3541, 3542, 3544, 3625, and 3626 on the other side. The sample No.3543 shows extreme values which are due to the high degree of biodegradation of steranes and terpanes as documented by the mass fragmentograms shown in Figure 9. Reference to types of potsherd and amphorae in Figure 10 show that there is no relationship between the type of bitumen and the type of potsherd.

**Figure 11:**
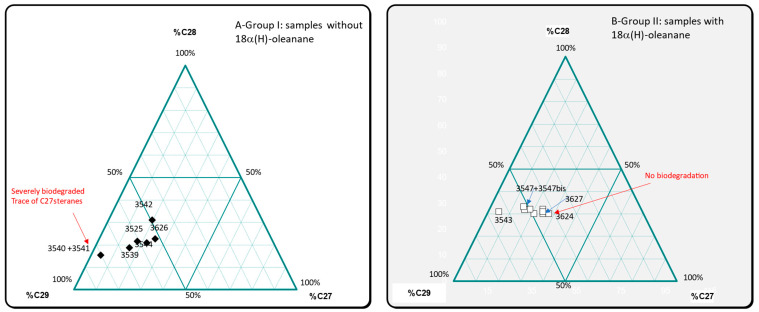
Plot of αββsteranes in a ternary diagram: %C_27_ vs. %C_28_ vs. %C_29_. (**A**) Group I: samples without 18α(H)-oleanane. (**B**) Group II: samples with 18α(H)-oleanane.

The plot of %C_27_αββsteranes vs. %C_28_αββsteranes vs. %C_29_αββsteranes in a ternary diagram (Figure 11), used to determine the origin of bitumens, confirms the two main groups of samples already defined: group I with samples No. 3539, 3540 + 3541, 3542, 3544, 3525, and 3526 originating from the Kermanshah province and group II with samples No.3543, 3545, 3545bis, 3546, 3547, 3547bis, 3623, 3624, and 3627 sourced from the Khuzestan and Fars provinces. Sample No.3542, which has a particular behavior, falls within group II.

The plot of diasteranes/regular steranes vs. steranes/terpanes or vs. C_24_tetracyclic terpane/C_23_tricyclic terpanes confirms the identified two groups of samples.

**Figure 12:**
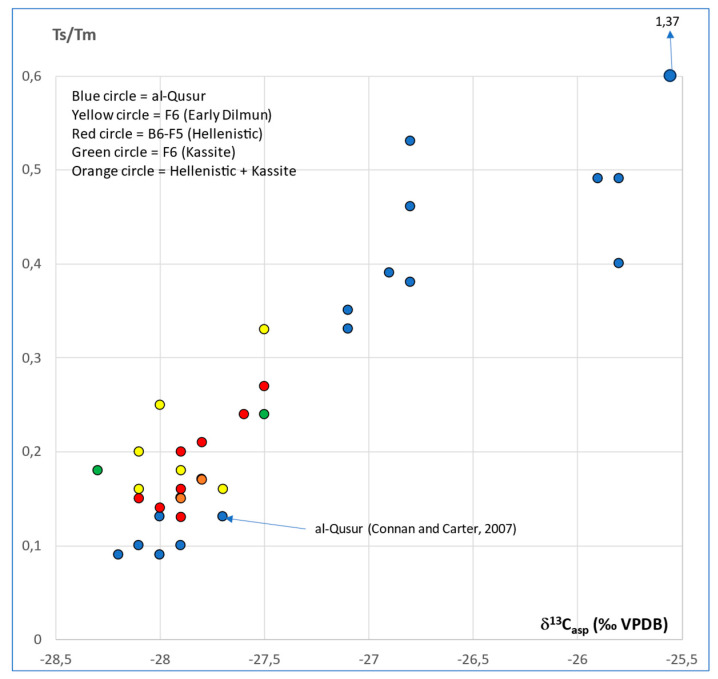
Plot of Ts/Tm vs. δ^13^Casp (‰/VPDB): comparison of al-Qusur to other sites of Failaka Island.

The plot of Ts/Tm vs. δ^13^C_asp_ (‰/VPDB) of al-Qusur data with other data already published [19] on other sites (F5, F6, and B6) from Failaka Island clearly differentiate al-Qusur from other sites (Figure 12). These sites covered other periods: Hellenistic (B6 and F5), Kassite (F6), and Early Dilmun (F6), and bitumens came from Iraq and Kuwait.

### 3.4. Detailed Geochemical Study of Samples No.3545 and 3545bis 

Large bitumen coated basins (SPORC) were identified within room R8 of building B20, in the last occupation layer of this food-processing workshop, dated between the end of the 8th and the beginning of the 9th century (Figure 1). These jars stand out from the rest of the ceramic corpus in terms of their shape and size. A functional study was carried out by combining all the data available prior to the physico-chemical analysis. The presence of numerous ichthyological remains in the adjoining room, engravings in the shape of fish on the body of one of the jars, the presence of bitumen, and a base studded with perforations suggesting a filtration system all point to the manufacture of ‘*murri’*. This medieval recipe, similar to ancient *garum*, used small, dried fish, wine, and various herbs and spices. This hypothesis was based on textual sources linking the consumption of this condiment to Christian communities [3].

**Figure 13:**
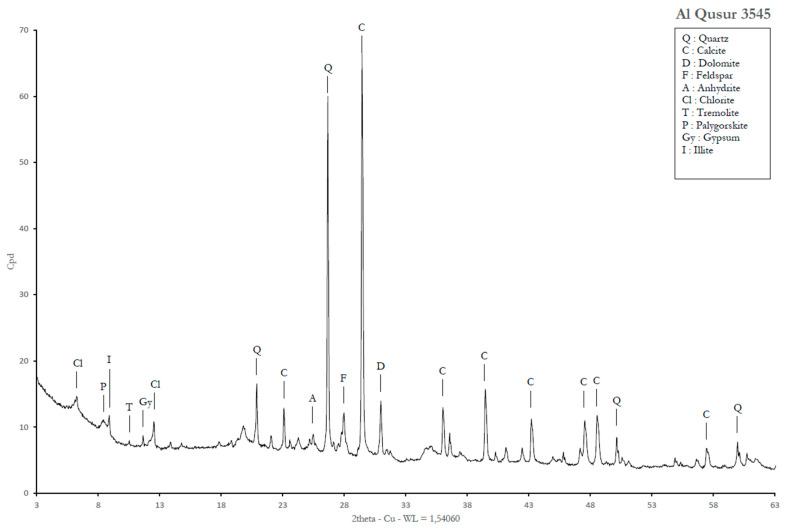
X-Ray diffraction diagram: identification of minerals in the sample No.3545.

The sample that we analyzed in duplicate (No.3545 and 3545bis) came from the larger basin, which seems to be a kind of juice strainer to filter the «*garum-like juice*». The mineralogical analysis carried out by Renaud Gley at the University of Lorraine (France) did not find evidence for any halite (NaCl), but classical mineral additives of archaeological bituminous mixtures [18]: quartz, calcite, and dolomite as the dominant minerals and calcium sulfates (gypsum and anhydrite) were often present in carbonates lithologies (Figure 13). In addition, clays (illite) and plagioclases (albite) were also identified.

The sample was analyzed by Philippe Schaeffer, University of Strasbourg, for complementary organic analyses.

**Figure 14:**
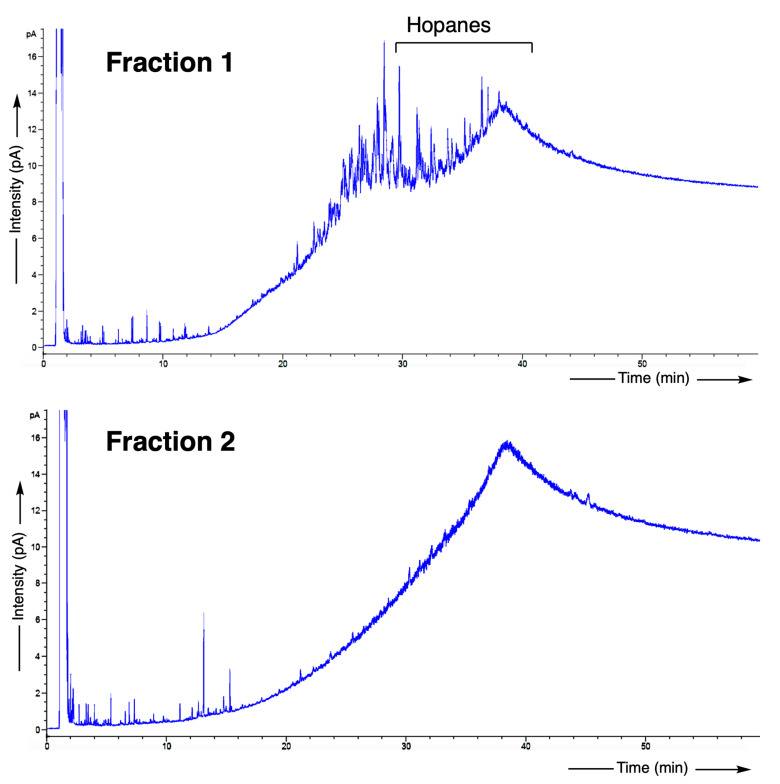
Gas chromatogram of the chromatographic fractions: F1 (hydrocarbons) and F2 (fatty acids and alcohols, analyzed as acetate and methyl ester derivatives, respectively) of the sample No.3545.

The mixture has been subsequently fractionated into two fractions: hydrocarbons and then alcohols and methyl esters. The gas chromatograms of the two fractions are shown in Figure 14. The hydrocarbon fraction F1 shows a distribution typical for a biodegraded bitumen (abundant unresolved complex mixture, lack of *n*-alkanes and isoprenoids, abundant hopane family). The fraction F2 also shows an unresolved complex mixture associated with biodegraded bitumen. This fraction has also been analyzed by GC-MS in order to try to detect diagnostic compounds: fatty acids, terpenoids, for instance. However, no specific compounds could be identified. As a consequence, it seems that the bitumen matrix has not adsorbed any molecules from the presumed “*garum*” filtered in the basin.

Although there are many arguments in favor of the production of ‘*murri’*, the absence of organic markers linked to fish products means that an alternative should be carried out.

## 4. Conclusions

The geochemical analysis of the 13 samples has shown the occurrence of bitumen in all samples.

This bitumen has different origins: Kermanshah province and Khuzestan, Fars, and Busher provinces in Iran.

The coating of the big basin with numerous holes (QSR16-A4/57) is composed mainly of bitumen and minerals, a typical archaeological bituminous mixture. No trace of the “*garum*” has been found within this mixture. It is interesting to note that whilst the archaeological evidence indicates the production of ‘*murri*’, the lack of organic biomarkers for fish opens the possibility for alternative uses.

## Figures and Tables

**Table 1:** Basic information on samples. The color refers to the type of potsherd.

Sample Number	Country	Lab Number	Location	Building	Context	Archaeological Reference	Latitude	Longitude	Date Range	Pottery Type
1	Kuwait	3539	al-Qusur	building B16 (monk’s cell)	5313	QSR-12-A5/18	29°26′12” N	48°20′52” E	Mid. 7th–early 9th CE	TORP-C
2		3540		building B20 (food processing), room R8	6067	QSR-16-A4/25			Late 8th–10th c. CE	CREAC (HAR-CR17) ?/BUFF ?
3		3541		building B20, room R8	6067	QSR-16-A4/25			Late 8th–10th c. CE	CREAC (HAR-CR17) ./BUFF ?
4		3542		building B14, courtyard	7103	QSR22-A5/0002			Mid. 7th–early 9th c. CE	TORP-S
5		3543		building B1	4	QSR07/2100			Mid. 7th–mid. 8th c. CE	TURQ.T (TUR-CR7)
6		3544		building B14, room R1	7162	QRS2022-A5/0001			Mid. 7th–early 9th c. CE	TORP-S
7		3545		building B20 (food processing), room R8	6067	QRS16-A4/57			Late 8th–10th c. CE	SPORC
8		3546		building B23 (refectory)	5635	QSR15-A4/243			Mid. 8th–10th c. CE	TORP-C
9		3547		building B23 (refectory)	5635	QSR15-A4/243			Mid. 8th–10th c. CE	TORP-C
**26**		3623		building B14, courtyard	7177	QSR23-A5-B14-7177			Mid. 7th–early 9th c. CE	TORP-S
27		3624		building B19, room R2	6854	QSR19-A5-U.97-6854			Mid. 7th–early 9th c. CE	TORP-S
28		3625		building B19, courtyard	6862	QSR19-A5-U.21-22-6862			Mid. 8th–early 9th c. CE	TORP-C
29		3626		building B20 (food processing), room R5	/	QSR23-A4-B20-U.63 R15			Mid. 8th–early 9th c. CE	TORP-C
30		3627		building B25, room R4	7701	QSR23-A6-B25-u.140-7701			/	/

**Table 2:** Gross composition of the dichloromethane extract (EO in % by weight/sample) and isotopic data: δ^13^C (‰/VPDB) and δD (‰/VSMOW = Vienna Standard Mean Ocean Water). Significance of abbreviations: sat = saturated hydrocarbons, aro = aromatic hydrocarbons, NSO= resins, asp = asphaltenes, pol = polars = resins + asphaltenes.

Sample Number	Country	Lab Number	Location	%EO	%Sat	%aro	%HC	%NSO	%ASP	%Pol	δ^13^Csat	δ^13^Caro	δ^13^CNSO	δ^13^Casp	δDNSO	δDasp
1	Kuwait	3539	al-Qusur	13.8	1.4	2	3.4	8.7	87.9	96.6	−28.5	−27.4	−28	−28	−84	−82
2		3540 + 3541		13.4	4.5	5.2	9.7	18.7	71.6	90.3	−27.4	−25.9	−28.1	−28.1	−91	−88
4		3542		53.5	3	4	7	9.5	83.5	93	−27.5	−26.4	−26.5	−27.1	−83	−74
5		3543		21	4	3.1	7.1	14.9	78	92.9	−27.1	−26	−26.1	−25.6	−87	−84
6		3544		15	1.7	3.2	4.9	12	83.1	95.1	−28.8	−27.3	−28.1	−28.2	−86	−71
7		3545		34.3	3.9	2.7	6.6	7.5	85.9	93.4	−27.2	−26.5	−26.9	−27.1	−85	−73
		3545bis		36.7	3.4	2.5	5.9	7.5	86.6	94.1	−27.6	−26.6	−26.8	−26.8	−82	−74
8		3546		22.1	3.3	3.5	6.8	7.1	86.1	93.2	−27	−25.9	−26.2	−25.8	−89	−69
9		3547		32.4	2.7	2.7	5.4	10.2	84.4	94.6	−27	−26	−26.3	−25.8	−108	−72
		3547bis		28.3	3.2	3	6.2	12.2	81.6	93.8	−27	−25.8	−26.5	−25.9	−101	−79
26	Kuwait	3623	al-Qusur	42.8	4.1	2.6	6.7	7.5	85.8	93.3	−27.5	−26.2	−26.8	−26.8	−80	−74
27		3624		41.5	4.5	2.2	6.7	5.3	88	93.3	−27.2	−26.3	−26.7	−26.9	−77	−77
28		3625		5.2	1.7	2.2	3.9	12.9	83.2	96.1	−27.2	−27.7	−28.2	−28	−103	−74
29		3626		1.7	1.4	1.4	2.8	8.4	88.8	97.2	−28.7	−27.8	−27.9	−27.9	−83	−72
30		3627		32.2	4.1	2.6	6.7	5.8	87.5	93.3	−27.4	−26.3	−26.8	−26.8	−81	−68

**Table 3:** Molecular data on steranes and terpanes. Significance of abbreviations: C_30_αβHopane = 17α,21β-hopane, OL/H = 18α(H)-oleanane/hopane, GA/C_31_R = Gammacerane/17α,21β,22R-30-homohopane, ster/terp = steranes/terpanes, Dia/reg = diasteranes/regular steranes, %C_27_αββR+S = 5α,14β,17β-20R+20S-cholestane,%C_28_αββR+S = 5α,14β,17β-20R+20S-24methylcholestane, %C_29_αββR+S = 5α,14β,17β-20R+20S-24ethylcholestane, C_29_ααα20S/20R = 5α,14α,17α-20S-24ethylcholestane/5α,14α,17α-20R-24ethylcholestane, C_29_H/C_30_H = norhopane/hopane, C_27_Ts/Tm = 18α-22,29,30-trisnorneohopane/17α-22,29,30-trisnorhopane, C_35_S/C_34_S = C_34_-17α,21β-22S-extended hopane/C_35_-17α,21β-22S-extended hopane.

Lab Number	C30αβHopane (ppm)	Tet/C23	C29/H	Ol/H	C31R/H	GA/C31R	GA/C30H	C35S/C34S	Ster/Terp	Dia/Reg	%C27	%C28	%C29	C2920S/20R	C29αββS/C29αααR	Ts/Tm	Tricyclics	Terpanes	Steranes	C27diasteranes
**3539**	**5525**	1.27	1.02	0	0.32	0.59	0.19	1.14	0.04	0.12	15.5	18.8	65.7	0.55	0.68	0.09	almost absent	well preserved	biodegraded -C27 < C28 < C29	
**3540 + 3541**	**13,768**	3.41	1.19	0	0.23	0.56	0.13	0.87	0.08	0.08	4.3	15.5	80.2	0.38	0.81	0.1	almost absent	preserved?	severely biodegraded-C27	
**3542**	**3428**	4.15	1.35	0	0.3	0.55	0.17	1.03	0.13	0.19	19.5	31.2	49.3	0.71	0.99	0.33	almost absent	preserved?	biodegraded	
**3543**	**109**	0.53	1.65	3.72	0.43	1.07	0.46	3.7	0.72	0.28	4.8	30.9	64.4	11.38	2.6	1.37	absent	severely biodegraded	severely biodegraded-	
**3544**	**10,580**	3.44	0.94	0	0.27	0.58	0.16	1.08	0.09	0.04	22.3	21	56.8	0.65	1.06	0.09	absent	preserved	slightly biodegraded	
**3545**	**3563**	1.15	0.98	0.1	0.31	0.36	0.11	1.15	0.36	0.69	25.1	29.9	45	0.76	1.37	0.35	present low	preserved	preserved	present
**3545bis**	**3426**	1.79	1.01	0.11	0.33	0.35	0.12	1.25	0.33	0.86	21.1	30	48.9	1.05	1.52	0.38	present low	preserved	preserved	present
**3546**	**1148**	1.26	1.01	0.35	0.32	0.36	0.12	1.72	0.75	0.75	18.3	32	49.7	1.18	1.71	0.4	present low	preserved	preserved	present
**3547**	**1569**	1.24	0.94	0.2	0.38	0.29	0.11	1.26	0.36	0.92	15.9	31.8	52.2	1.19	1.71	0.49	present low	preserved	slightly biodegraded-C29αββR -C27αββR biodegraded	present
**3447bis**	**2027**	1.85	0.94	0.17	0.36	0.3	0.11	1.19	0.34	0.87	14.8	33.1	52.1	1.16	1.79	0.49	almost absent	preserved	slightly biodegraded-C29αββR -C27αββR biodegraded	present
**3623**	1464	1.1	1.19	0.16	0.34	0.34	0.12	1.3	0.82	1.09	24.1	32	43.9	1.28	2.05	0.46	almost absent	preserved	slightly biodegraded-C29αββR -C27αββR biodegraded	present
**3624**	4313	1.46	1.06	0.1	0.32	0.31	0.1	1.05	0.33	0.81	27.8	30	42.2	0.88	1.42	0.39	almost absent	preserved	preserved	abundant
**3625**	2786	2.14	1.04	0	0.35	0.73	0.26	1.18	0.04	0.59	17.5	21.8	60.7	0.71	0.74	0.13	absent	preserved	biodegraded-C27 < C28 < C29	present low
**3626**	21,510	3.62	1.13	0	0.25	0.54	0.14	0.9	0.08	0.05	24.9	22.9	52.2	0.47	0.77	0.1	absent	preserved	biodegraded-C27 < C28 < C29	absent
**3627**	1244	0.58	1.23	0.17	0.32	0.42	0.13	1.55	0.7	1.09	24.6	30.8	44.6	1.18	1.44	0.53	present	preserved?	preserved	abundant

## Data Availability

Data are contained within the article.
